# Multilocus Comparative Phylogeography of Two Aristeid Shrimps of High Commercial Interest (*Aristeus antennatus and Aristaeomorpha foliacea*) Reveals Different Responses to Past Environmental Changes

**DOI:** 10.1371/journal.pone.0059033

**Published:** 2013-03-13

**Authors:** Maria Victoria Fernández, Sandra Heras, Jordi Viñas, Ferruccio Maltagliati, Maria Inés Roldán

**Affiliations:** 1 Laboratori d’Ictiologia Genètica, Departament de Biologia, Universitat de Girona, Campus de Montilivi, Girona, Spain; 2 Dipartimento di Biologia, Università di Pisa, Pisa, Italy; University of Poitiers, France

## Abstract

Phylogeographical studies can reveal hidden patterns in the evolutionary history of species. Comparative analyses of closely related species can further help disentangle the relative contributions of processes responsible for such patterns. In this work, the phylogeography of two aristeid species, *Aristeus antennatus* and *Aristaeomorpha foliacea*, was compared through multiple genetic markers. These marine shrimp species are of high commercial importance, and are exploited in the Mediterranean Sea (MED) and in Mozambique Channel (MOZ) where they occur in partial sympatry. *Aristeus antennatus* (N = 50) from Western and Eastern Mediterranean (WM and EM, respectively), Atlantic Ocean (AO) and MOZ, and *Aristaeomorpha foliacea* (N = 40) from WM, EM, MOZ North-Western Australia (AUS) were analyzed with two nuclear genes (PEPCK and NaK) and one mitochondrial (COI) gene. Within the study area differences were found between the two species in their phylogeographical patterns, suggesting distinct responses to environmental changes. Monophyly of *Aristeus antennatus* was found across its distributional range. This pattern contrasted by a deep evolutionary split within *Aristaeomorpha foliacea* where genetic diversity followed geography distinguishing MED-MOZ and AUS. We propose that the AUS lineage of *A. foliacea* warrants consideration as a distinct species, with consequent implications in systematics and resource management.

## Introduction

Aristeid shrimps (Aristeidae, Wood-Mason 1891) are a group of commercially important species within the superfamily Penaeoidea (Rafinesque 1815), which are also known as red shrimps because of their body coloration [1]. *Aristeus antennatus* (Risso, 1816) and *Aristaeomorpha foliacea* (Risso, 1827) are the two most economically valuable species of the Aristeidae family. They occur in sympatry in a large part of their geographical and bathymetrical distribution. *Aristeus antennatus* can be found between 80 [2] and 3300 m depth [3], whilst *Aristaeomorpha foliacea* is distributed between 120 and 1000 m depth [4]; yet both species present their maximum abundance levels between 400 and 800 m depth [3], [4]. *Aristeus antennatus* is distributed in the Mediterranean Sea and adjacent Atlantic Ocean, from Portugal to Cape Verde Islands [5]. In the Indian Ocean, its distribution is restricted to the Maldives Islands, Zanzibar Island, Mozambique and South Africa [5]. *Aristaeomorpha foliacea* is found within the same geographical range of *Aristeus antennatus*, but in the Indian Ocean its distribution is much wider, reaching North-Western Australia coastal waters [6]. Since 1930s both shrimp species have been harvested in the Mediterranean Sea, constituting an important directed fishery that currently represents 30% of the local income of many local Mediterranean ports [7]. Recently, a second major area of exploitation for red shrimps has been established in the Mozambique Channel [8]. Minor harvesting areas also exist off the coast of Portugal for *Aristeus antennatus* [9] and off North-Western Australia for *Aristaeomorpha foliacea* [6].

Despite the high economic value of both species, little is known about their systematics, or their biology, ecology and population genetics outside the Mediterranean Sea. Information on phylogenetic relationships within Aristeidae and with other Penaeoidea families is very scarce. Only two surveys included aristeid species as a part of reviews on the phylogeny of Decapoda (*Aristeus virilis*) [10], and the phylogeny of Penaeoidea (*Aristaeomorpha foliacea*) [11]. From a population genetic perspective, a number of studies on *Aristeus antennatus* and *Aristaeomorpha foliacea* were consistent in showing a general picture of relative genetic homogeneity within the Mediterranean Sea [12]–[16]. Genetic analysis including samples beyond the Mediterranean basin detected moderate to high levels of genetic differentiation in both species [17], [18]. For example, mitochondrial DNA (mtDNA) analysis of *Aristeus antennatus* detected significant genetic differentiation among Western Mediterranean, Eastern Mediterranean, Atlantic Ocean and Mozambique Channel [17]. Similarly, analysis of *Aristaeomorpha foliacea* detected three mtDNA monophyletic lineages corresponding to Mediterranean Sea, Mozambique Channel and North-Western Australia [18].

Given their close taxonomic relationship, partial sympatry and large similarities in adult ecology and reproductive biology [19], these two shrimp species are ideal candidates for a study of comparative phylogeography. Such studies should be based on diverse loci because different genes may be responding idiosyncratically to the evolutionary forces operating upon populations [20]. A major limitation of previous genetic studies on these two species [17], [18] is their reliance on mtDNA which only partially reflects their evolutionary history. In addition to one mitochondrial marker (cytochrome *c* oxidase subunit I, COI), we include nuclear molecular markers in this study: phosphoenolpyruvate carboxykinase (PEPCK) and sodium–potassium ATPase α -subunit (NaK). These markers have proven satisfactory to resolve phylogenies of crustacea and insecta [10 and references therein].

Our objective was to compare the phylogeographical patterns of *Aristeus antennatus* and *Aristaeomorpha foliacea* to contrast how these two species have been influenced by present or historical factors. Such comparisons can provide sound information on the evolutionary history of species, as well as help in the identification of evolutionary isolated areas and unveil hidden cryptic species [21]. The information provided here should prove useful, with implications for conservation and management of these important commercial species as well as to gain insight into the phylogenetic relationships within and between *A*. *antennatus* and *A. foliacea.*

## Methods

### Sampling

Sub-samples of 50 *Aristeus antennatus* and 40 *Aristaeomorpha foliacea* individuals were selected from previous works based on mtDNA [17], [18] according to two criteria: i) each putative biogeographical region had to be represented by 10 individuals, ii) previous levels of within locality mitochondrial genetic were maintained (Table S1 and Table S2). Biogeographical regions selected for *Aristeus antennatus* [17] were Alborán Sea (ALB), Western Mediterranean (WM), Eastern Mediterranean (EM), Atlantic Ocean (AO) and Mozambique Channel (MOZ) (Table 1); Gulf of Lion was selected as representative of WM and Ionian Sea as representative of EM. It has been inferred that *A. antennatus* between the Ionian Sea and Aegean Sea are genetically similar based on the fact that *A. foliacea* were genetically similar between these two Seas [18]. The biogeographical regions selected for *Aristaeomorpha foliacea* [18] were WM, EM, MOZ and North-Western Australia (AUS); Cabrera was selected as representative of WM and Aegean Sea as representative of EM (Table 1). The Strait of Sicily was considered as the seamark to separate EM, WM following Millot [22]. Finally, three individuals of *Aristeus virilis* were also included in the analysis (collected in Mozambique Channel, MOZ0308 survey by the *Instituto Español de Oceanografia*) for comparative purposes. Available GenBank sequences for the three studied genes were found only for *Penaeus monodon* (COI: PRJNA11894, PEPCK: EU427213, NaK: EU427144) and *Solenocera crassicornis* (COI: AY264902, PEPCK: FJ441211, NaK: FJ441166), which belongs to the same superfamily (Penaeoidea), and were used as outgroup species in phylogenetic analyses.

**Table 1:** Estimates of genetic diversity obtained for each molecular marker employed.

Species	CODE	Geographicalcoordinates	COI (514 bp)					PEPCK(536 bp)			NaK(498 bp)		
Biogeographical region			n	nh	*h ±* S.D.	np	π *±* S.D.	n	F	*H*_O_	n	F	*H*_O_
*Aristeus virilis*	Av												
Mozambique Channel	MOZ	17° 36′ S, 38° 26′ E	3	3	1.000±0.074	2	0.0026±0.002	3	2	1	2	2	0.5
*Aristeus antennatus*	Aa												
Alborán Sea	ALB	35° 59′ N, 03° 05′ W	10	4	0.533±0.180	6	0.0028±0.002	10	4	0.40	5	9	0.80
Western Mediterranean	WM	42° 35′ N, 04° 13′ E	10	3	0.378±0.181	5	0.0019±0.002	6	3	0.16	5	9	0.75
Eastern Mediterranean	EM	37° 37′ N, 21° 03′ E	10	5	0.800±0.100	12	0.0072±0.004	7	4	0.57	7	12	0.86
Atlantic Ocean	AO		10	7	0.911±0.077	11	0.0052±0.004	9	4	0.44	2	4	1
Mozambique Channel	MOZ	17° 32′ S, 38° 29′ E	10	9	0.978±0.054	12	0.0062±0.004	10	4	0.50	8	16	1
Total *A. antennatus*			50	17	0.777±0.059	24	0.0051±0.001	42	4	0.43	27	38	0.88
*Aristaeomorpha foliacea*	Af												
Western Mediterranean	WM	39° 02′ N, 02° 39′ E	10	5	0.756±0.130	4	0.0022±0.002	10	10	0.70	7	4	1
Eastern Mediterranean	EM	37° 17′ N, 22° 53′ E	10	4	0.644±0.152	4	0.0032±0.002	10	10	0.60	8	2	1
Mozambique Channel	MOZ	25° 57′ S, 34° 38′ E	10	5	0.667±0.163	5	0.0019±0.002	10	6	0.50	10	4	1
North-Western Australia	AUS	14° 51′ S, 121° 26′ E	10	10	1.000±0.045	12	0.0614±0.004	9	5	0.60	5	2	1
Total *A. foliacea*			40	20	0.927±0.022	47	0.0311±0.004	39	12	0.62	30	6	1

Number of individuals (n), number of haplotypes (nh), number of polymorphic sites (np), haplotype (*h*) and nucleotide (π) diversity with standard deviation (S.D.) of mtDNA COI; number of alleles (F), observed heterozigosity (*H*_O_ *)* of nuclear loci PEPCK and NaK.

### DNA Extraction, PCR Amplification and Sequencing

DNA extraction of ethanol-preserved samples, polymerase chain reaction (PCR) and sequencing of COI followed the procedures outlined in Fernández et al. [18]. Amplification of PEPCK was performed with primers described in Tsang et al. [10]. New primers for NaK amplification were designed based on 48 Penaeoidea sequences available in GenBank [10], [11]. Final primers were NaK-fAr (5′-TGGCTGCCAGTATGSCAAGA-3′, for Aristeidae), NaK-rAa (5′-CGGAGGATCAATCATCGACA-3′, for *Aristeus* spp.), NaK-rAf (5′-CGGAGGATCAATCATGGACA-3′, for *Aristaeomorpha foliacea*). Amplifications for PEPCK and NaK were carried out in a reaction mix containing 1–3 µl of template DNA (25 ng µl^−1^), 1X PCR reaction buffer, 3 mM MgCl_2_, 200 µM dNTPs, 200 nM of each primer, and 0.03 U of DNA polymerase (Ecotaq, Ecogen) in a 30 µl final volume. The PCR profile for both nuclear genes was as follows: 3 min at 94°C for initial denaturation, followed by 35 cycles of 30 s at 94°C, 30 s at 57°C and 55°C annealing temperatures for PEPCK and for NaK, respectively, 90 s at 72°C with a final extension for 10 min at 72°C. Non-template controls were run in all PCRs to ascertain that no cross contamination took place. PCR products were verified on 1% agarose gel with ethidium bromide (0.5 mg/ml). Sequences were cleaned for sequencing by treating with exonuclease I and shrimp alkaline phosphatase [23]. DNA sequencing reactions were carried out using BigDye Terminator v3.1 Cycle Sequencing Kit (Applied Biosystems) according to the manufacturer’s instructions and read in an ABI PRISM 3130 Genetic Analyzer (Applied Biosystems) at the *Laboratori d’Ictiologia Genètica*, *Universitat de Girona*, Spain.

### Sequence Data Analysis

Nucleotide sequences were aligned and edited in SeqScape v2.5 (Applied Biosystems) employing as reference the partial regions of COI, PEPCK and NaK genes from *Aristaeomorpha foliacea* (GenBank accession numbers: JN676306, FJ441125 and FJ441170, respectively). Final edition and concatenation of the three genes were performed with BioEdit v7.0.4.1 [24]. The nuclear molecular markers amplicons were located on the exons of the genes, as indicated in Friedlander et al. [25], [26], no indels were observed and ambiguous nuclear positions (i.e. double peaks in the chromatogram, corresponding to putative heterozygote sites) were coded using IUPAC symbols for all subsequent analyses, except for heterozygosity calculations. PHASE algorithm [27], [28], as implemented in DnaSP v5 [29], was used to reconstruct putative alleles of each nuclear gene (coded as Allele 1 and Allele 2 in Table S2).

Genetic variability estimates were calculated on each gene, separately. Haplotype and nucleotide diversity were calculated for COI gene with DnaSP v5, and expected heterozygosity was obtained for PEPCK and NaK genes with the online version of Genepop v4.0.10 [30] after haplotype reconstruction. A chi-squared (χ^2^) statistic, with Bonferroni correction [31], was calculated from a contingency table in which one dimension consists in the frequency of nuclear alleles and the second dimension consists of biogeographical regions, in order to evaluate the homogeneity in the distribution of nuclear allele frequencies between biogeographical regions.

A partition homogeneity test [32] was carried out with PAUP* v4.0 b10 [33], in order to assess the correctness of using the concatenated dataset for phylogenetic inference. The program jModelTest v0.1 [34] was used to run a hierarchical series of tests based on the Akaike Information Criterion (AIC) to identify the best-fit model of nucleotide substitution for the concatenated dataset of all the species studied considered among 88 models tested. The model selected was TN [35] with α = 0.566, *i* = 0.563, and base frequency A = 0.261, C = 0.255, G = 0.215, T = 0.269.

Maximum likelihood (ML) [36] and Neighbor-Joining (NJ) [37] analyses were conducted in PAUP. NJ was based on Tamura-Nei genetic distances [35]; ML was performed with an heuristic search and tree bisection-reconnection branch-swapping algorithm, 100 replicates and “as-is” was chosen for sequence addition. Robustness of trees was tested using bootstrap analyses [38] with 1000 replicates. Furthermore, MrModeltest v2.3 [39] was used to estimate best evolutionary model under Bayesian Inference (BI) analysis. The model selected was SYM [40] with α = 0.691, *i* = 0.576, and equal base frequencies; successively, a Bayesian phylogenetic tree was constructed with MrBayes v3.1.2 [41] with metropolis-coupled Markov Chain Monte Carlo algorithm. Four replicate runs were carried out with the value of four Markov chains per run for 2 x 10^6^ generations. The chain was sampled every 100 generations to obtain 20 000 sampled trees. The first 5 000 trees (25%) were discarded as the burn-in phase. A final consensus tree with branch length and clade credibility (posterior probability) was generated with the 75% remaining samples.

Analysis of molecular variance (AMOVA) [42] and Φ-statistics for concatenated dataset were conducted with Arlequin v3.5 [43]. Significance of Φ-statistics was estimated by a permutation test with 10 000 pseudoreplicates. This analysis was used to partition genetic variance in the among- and within-sample components for *Aristeus antennatus* and *Aristaeomorpha foliacea* datasets. Tamura-Nei genetic distances [35], with a gamma distribution (α = 0.566) and considering the composition bias among sequences of concatenated dataset, were calculated between lineages detected by phylogenetic trees (ML; NJ; BI). Genetic distances between lineages were also calculated for each molecular marker separately following the models selected by JModeltest v0.1 [34] based on AIC. TN [35] with *i* = 0.69 for COI, TN [35] with *i* = 0.636 and α = 0.466 for NaK, and K2P [44] with *i* = 0.926 for PEPCK. Standard errors were obtained after 10 000 replicates. Correction between groups for all genes was calculated (*D*_A_) [45]. All genetic distances were calculated with MEGA v5 [46]. Evolutionary relationships among phylogenetic groups from concatenated and mitochondrial dataset was inferred by constructing a NJ tree after 10 000 replicates. A median-joining network of COI haplotypes was constructed using NETWORK v4.600 [47].

## Results

From a total of 50 *Aristeus antennatus*, 42 individuals were successfully sequenced for PEPCK (536 bp) and 27 for NaK (498 bp) gene, providing five and 26 different genotypes, respectively (Table S2). From a total of 40 *Aristaeomorpha foliacea*, 39 amplified for PEPCK and 30 for NaK gene, providing 20 and 6 different genotypes, respectively. The analysis of three *Aristeus virilis* provided three different COI (514 bp) haplotypes plus one PEPCK and two NaK different genotypes. Sequences were deposited in GenBank (Table S2). The partition homogeneity test did not reveal incongruence between molecular markers (*P = *0.194) allowing their combination for successive analyses. The final concatenated dataset consisted of 55 sequences (23 *Aristeus antennatus,* 30 *Aristaeomorpha foliacea* and 2 *Aristeus virilis*) and the two outgroup sequences that presented 236 parsimony informative sites from a total of 1 548 bp.

### Genetic Diversity

The comparative analysis of mitochondrial COI gene indicated that both species presented high levels of haplotypic diversity (*h* >0.378), with the highest values for *Aristeus antennatus* and *Aristaeomorpha foliacea* in MOZ and AUS, respectively (Table 1). Within the geographical area where these two species are co-distributed, *Aristeus antennatus* exhibited levels of haplotypic and nucleotidic diversity in MOZ and EM higher than those obtained for *Aristaeomorpha foliacea*. Conversely, in WM *Aristaeomorpha foliacea* exhibited haplotype diversity higher than that of *Aristeus antennatus*, although nucleotide diversity values were similar (Table 1). Private haplotypes in all geographical regions were detected for both species (Table S2).

Genetic variability of PEPCK was higher in *Aristaeomorpha foliacea*, which showed 12 different alleles, whilst *Aristeus antennatus* exhibited only four (Table 1 and Table S2). This outcome is also reflected by the heterozigosity values, which are higher in *Aristaeomorpha foliacea* (*H*_O_ *= *0.62) than in *Aristeus antennatus* (*H*_O_ *= *0.43) (Table 1). Each species was clearly distinguished by their genotypes. *Aristeus antennatus* did not exhibit region specific genotypes, but *Aristaeomorpha foliacea* presented private genotypes in MED (Af-ph1, Af-ph2, Af-ph3, Af-ph7, Af-ph8, Af-ph12, Af-ph13, Af-ph16) and AUS (Af-ph20), and some alleles of those genotypes (Allele 11, 12, 13, 16) were private for regions as well (Table S2).

NaK genetic variability was higher in *Aristeus antennatus* than in *Aristaeomorpha foliacea*, as revealed by the higher number of alleles detected in the former species (38 *vs*. 6) (Table 1 and Table S2). However, observed heterozygosity values were higher in *Aristaeomorpha foliacea*, in which all individuals were heterozygotes (*H*_O_ = 1), than in *Aristeus antennatus* (*H*_O_ *≥*0.75) (Table 1). Almost each individual of *Aristeus antennatus* exhibited a different genotype, with the exception of genotype Aa-nh3, which occurred in WM and EM; hence, no geographical association could be drawn (Table S2). Instead, relationships between NaK genotypes and geographical origin of samples were detected in *Aristaeomorpha foliacea*; all of AUS individuals expressed a private genotype (genotype Af-nh6, Table S2) due to the presence of a private allele (allele 44, Table S2) and MOZ showed two private genotypes (genotype Af-nh1 and Af-nh5, Table S2), and a private allele (allele 40, Table S2).

### Phylogeographical Analysis

**Figure 1:**
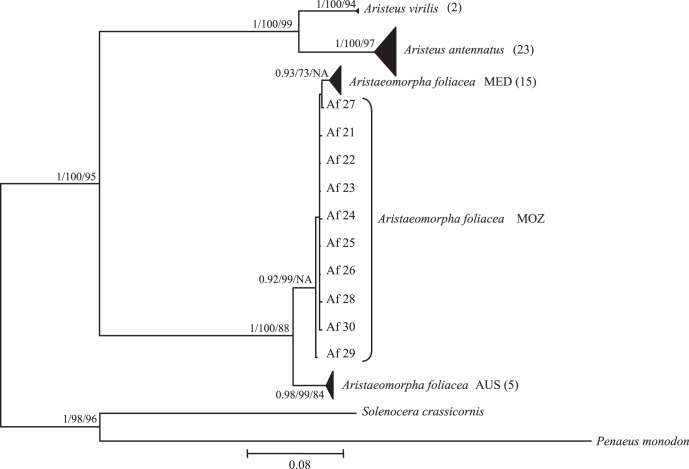
Bayesian condensed tree based on concatenated dataset. Tree was inferred from 57 sequences of concatenated COI, PEPCK and NaK fragments (1 548 bp). *Solenocera crassicornis* and *Penaeus monodon* were used as outgroup. The numbers on nodes indicate posterior probability values for Bayesian tree and bootstrap values for Neighbor-Joining and Maximum Likelihood trees respectively. Triangle sizes are proportional to the number of sequences present in the cluster (number in brackets). Location codes as in Table 1. The numbers on nodes indicate bootstrap values (≥70) after 10 000 replicates. NA: not available.

ML, NJ and BI analyses for the concatenated dataset generated identical tree topologies (Figure 1). Two major lineages corresponding to the *Aristeus* and *Aristaeomorpha* genera were identified. Within *Aristeus*, *Aristeus virilis* and *Aristeus antennatus* clustered into exclusively monophyletic lineages. Within *Aristeus antennatus* no clear associations between geographical distribution and sequences obtained was detected. Within *Aristaeomorpha*, two major phylogroups and geographical association of genetic diversity were detected. One group corresponding to AUS and the second including MED and MOZ, where MED appears monophyletic (Figure 1).

**Figure 2:**
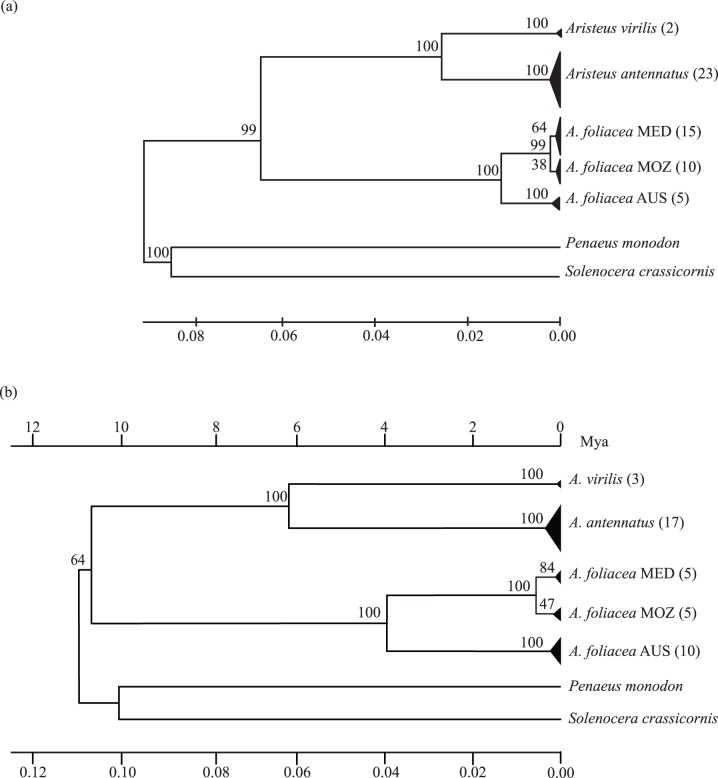
Neighbor-Joining trees based on Tamura-Nei genetic distances between biogeographical regions. (a) Condensed tree of genetic distances from concatenated loci of Table S3; (b) condensed tree of genetic distances from COI data of Table S5 with bar on top showing estimated time since divergence (Mya) using 1.015% as mean of 0.83–1.2% evolutionary rate. *Solenocera crassicornis* and *Penaeus monodon* were used as outgroups. The numbers on nodes indicate bootstrap values after 10 000 replicates. Triangle sizes are proportional to the number of sequences present in the cluster (number in brackets).

The lack of a clear geographical pattern in the distribution of genetic diversity in *Aristeus antennatus* was corroborated by the low and non-significant “among samples” component of molecular variance (9.6%, Φ_ST_ = 0.096, *P* = 0.079) (Table 2). Conversely, the high levels of genetic divergence detected in *Aristaeomorpha foliacea* (87.4% of variance among samples, Φ_ST_ = 0.874, *P*<0.001) supported the existence of genetic differentiation at the regional level (Table 2). The average within-species genetic distance from concatenated dataset was lower for *Aristeus antennatus* (*D* = 0.0033±0.0008) than that found in *Aristaeomorpha foliacea* (*D* = 0.0094±0.0015) (Table S3); net genetic distance between *A. foliacea* lineages, MED-MOZ and AUS, (*D*_A_ = 0.0226±0.0038) was about the half of genetic distance between *Aristeus* species (*D*_A_ = 0.0492±0.0062) (Table S3). Deep divergence of *Aristaeomorpha foliacea* lineages related to three biogeographical regions was also detected in the NJ tree based on genetic distances within and between species of Table S3 (Figure 2a).

**Table 2:** Analysis of molecular variance (AMOVA) for concatenated data (1 548 bp).

Species	Source of variation	df	Variance components	%	Φ-statistics	*P*
*A. antennatus*	Among samples	4	0.23459	9.57	Φ_ST_ = 0.096	0.079
	Within samples	18	2.21667	90.43		
*A. foliacea*	Among samples	3	7.70177	87.36	Φ_ST_ = 0.874	<0.001
	Within samples	26	1.11429	12.64		

Genetic distances from nuclear data (Table S4) among Aristeidae species of this study were higher for NaK (*D*_A_ = 0.0301±0.0064 to *D*_A_ = 0.1051±0.0171) than for PEPCK (*D*_A_ = 0.0056±0.0022 to *D*_A_ = 0.0238±0.0044). These values fall within the range previously reported by Ma et al. [11] between species of the family Aristeidae (NaK *D* = 0.01–0.099, PEPCK *D* = 0.002–0.036). Very low genetic distance values were obtained between MED and MOZ regions of *Aristaeomorpha foliacea*, which were then pooled together to estimate the genetic distance between MED-MOZ and AUS regions (NaK *D*_A_ = 0.0029±0.0013, PEPCK *D*_A_ = 0.0002±0.0001). The values of mitochondrial genetic distance between Aristeidae species (*D*_A_ = 0.1143–0.1949) were of the same order of magnitude of those values with outgroup species (Table S5 and Figure 2b). The values of genetic distance between *Aristaeomorpha foliacea* regions MED-AUS (*D*_A_ = 0.0690±0.0117) and MOZ-AUS (*D*_A_ = 0.0698±0.0117) are equivalent to the 61% genetic distance between true congeneric species (*Aristeus antennatus* and *Aristeus virilis*, *D*_A_ = 0.1143±0.0153); however, the genetic distance between MED and MOZ *Aristaeomorpha foliacea* geographical regions is equivalent to the 8% (*D*_A_ = 0.0090±0.0040) (Table S5).

**Figure 3:**
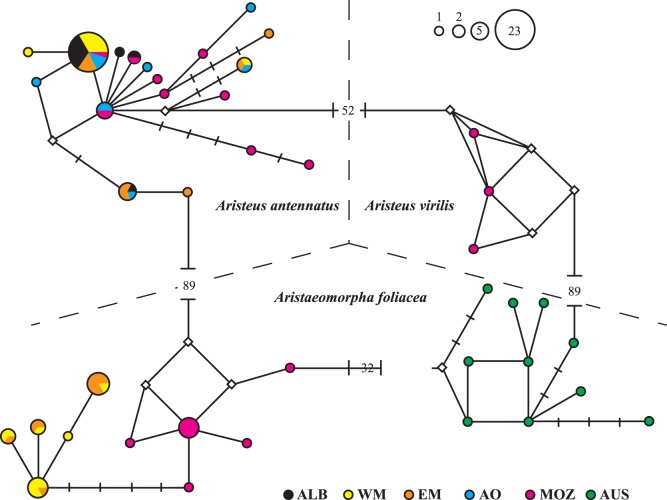
Median-joining network of COI haplotypes detected for the three species studied. The area of each circle is proportional to the number of individuals exhibiting that haplotype. Each line in the network represents one mutational step, vertical bars and white rhombi represent mutational steps and median vectors, respectively, both interpreted as missing or undetected haplotypes. Location codes as in Table 1.

The median-joining network of haplotypes clearly separated *Aristeus antennatus, Aristeus virilis* and *Aristaeomorpha foliacea* by a large number of mutational steps and connected the three species in a circle (Figure 3). *Aristeus antennatus* consisted of a single network connected to *Aristeus virilis* subnetwork by 52 mutational steps (MSs), whilst *Aristaeomorpha foliacea* presented three subnetworks, each corresponding to one of the regions considered. The subnetwork corresponding to MOZ connected with i) MED subnetwork through four MSs, ii) AUS subnetwork through 32 MSs, and iii) *Aristeus antennatus* through 89 MSs. The subnetwork corresponding to AUS connects with *Aristeus virilis* through 89 MSs.

## Discussion

Genealogical concordance is expected to be found among closely related taxa, particularly if they share the same habitat and are co-distributed [21]. However, instances of discordance in phylogenetic patterns among co-distributed closely related marine species have been described [48], [49]. This study provides another example of different evolutionary histories between two partially sympatric species.

### Genealogical Concordance within Species

Phylogeographical analysis of *Aristeus antennatus* showed concordant patterns across genes. Previous mitochondrial genetic analysis detected significant genetic differences between the Mediterranean Sea (MED), the Atlantic Ocean (AO) and the Mozambique Channel (MOZ) [17],; however the presence of common haplotypes among these regions (Figure 3) indicates that *A. antennatus* is a monophyletic lineage. This outcome is corroborated by *i*) the homogeneity in the distribution of nuclear alleles frequencies between regions (Table S6) and *ii*) the combined analyses of mitochondrial and nuclear genes carried out in the present work (Φ_ST_ = 0.096, *P* = 0.079) (Figure 1 and Figure 2). Conversely, phylogeographical analysis of *Aristaeomorpha foliacea* showed discordant genetic partitions across multiple and independent (mitochondrial and nuclear) loci. Previous mtDNA genetic analysis detected three highly differentiated lineages that were geographically characterized: MED, MOZ and North-Western Australia (AUS) [18]. The combined mitochondrial and nuclear markers employed in the present study corroborated that mitochondrial signature of genetic divergence (Φ_ST_ = 0.874, *P*<0.001), confirming the existence of a clearly differentiated AUS lineage (Figure 1). The mitochondrial reciprocal monophyly detected between MED and MOZ (Figure 3) was not fully supported by nuclear markers, based on measures of genetic distance (Table S4), which placed the individuals of these two regions within the same node (Figure 1). However, the distinction of these two regions was supported by significant divergence of allele frequencies at nuclear loci (Table S6). Incomplete lineage sorting of nuclear markers may account for such results. If a matrilinear tree for two isolated populations has marginally achieved a status of reciprocal monophyly, then about 3× more time is required for a typical nuclear gene to achieve the same status through lineage sorting [50]. Consequently, not enough time would have passed for the mitochondrial monophylies of MED and MOZ to be reflected in the nuclear intraspecific phylogeny.

### Genealogical Discordance between Species

Discordant patterns detected within the regions in which *Aristeus antennatus* and *Aristaeomorpha foliacea* co-occur (MED and MOZ) prompted the following hypotheses: i) a vicariant event separated the MED and MOZ populations of these species, where ii) evolutionary forces and/or ecological processes had a lesser effect on *A. antennatus* than on *A. foliacea*, shaping intraspecific phylogenies differently. These hypotheses are discussed below.

Based on COI genetic distances and using 0.83–1.2% evolutionary rate for COI gene (as reviewed in Ketmaier et al. [51], the divergence between MED and MOZ regions of *Aristaeomorpha foliacea* has been estimated at ca. 0.5 Mya (Table S5). The Benguela Current (BC) upwelling system is now considered a major barrier for many marine organisms between eastern and western South African coasts [52]. The final closure of the Isthmus of Panama provoked changes in ocean circulation and marked the transition to a period of cold climate worldwide. As a result, the BC responded with a pronounced upwelling system at 2.1–1.9 Mya with further intensifications during Pleistocene glacial cycles at ∼ 0.6 Mya. Consequently, average surface temperature lowered from the 26°C in the mid-Pliocene (3.5 Mya) to approximately 18°C in modern times [53]. The intensification of the BC upwelling system could have acted as vicariant event causing the disappearance of *Aristeus antennatus* and *Aristaeomorpha foliacea* within its area of influence (between Cape Verde and South Africa) where currently there is no knowledge of their presence [5]. However, because of their relatively thin cuticula, shrimps tend to be underepresented in the fossil record [1], preventing testing of this vicariant hypothesis.

After the vicariant event, MED and MOZ would have evolved independently and Pleistocene climatic oscillations would have played an important role in the shaping of different evolutionary histories in *A. antennatus* and *A. foliacea*, possibly due to different life-history traits. For example, it has been proposed that *Aristaeomorpha foliacea* would be more sensitive to changes in environmental conditions, due to its higher susceptibility to low levels of dissolved oxygen in the water [54], implying a greater susceptibility to Pleistocene climatic changes than *Aristeus antennatu*s. In contrast, because the water column acts as a natural buffer against climatic oscillations, deep water masses remain more stable than superficial waters [55]. Since *Aristeus antennatus* occurs at greater depths than *Aristaeomorpha foliacea*, the former species could have found refugia in deeper waters during glacial cycles. During these severe climatic oscillations *Aristaeomorpha foliacea* populations would have suffered recurrent cycles of reduction and expansion of its populations. The low effective size of *A. foliacea* populations would have made this species more susceptible to stochastic changes in genetic composition than the larger and more stable *A. antennatus* populations where MED and MOZ share haplotypes. Clearly these hypotheses need further investigation.

### Interspecific Phylogeny and Speciation

This study showed the close relationship between *Aristeus virilis* and *Aristeus antennatus* based on multilocus analyses which is consistent with congeneric species level (Figure 1, Table S4). Within *A. antennatus*, a unique lineage was defined throughout the study area, which covers most of its spatial distributional range. In contrast, two clearly distinguished monophyletic lineages of *Aristaeomorpha foliacea* were detected, whose genetic distance (*D*_A_ = 0.0226±0.0038) was almost the half of that detected between true congeneric species (Table S3).

Through the speciation process, divergent lineages undergo changes in genotypic and phenotypic properties that lead to morphological differentiation, reproductive isolation and ecological differentiation; such changes neither occur at the same time, and nor necessarily in a regular order [56]. Therefore, when gene flow is restricted between lineages for a long time, reproductive isolating mechanisms (RIMs) and morphological differences will eventually appear [56]. The inability to test the Biological Species Concept (BSC) [57] in this deep-sea marine species does not imply that both lineages have not developed RIMs. Also, it is well known that decapods and particularly penaeids species evolve large genetic differences with apparently no morphological variability [58]–[61]. Palumbi & Benzie [60] proposed a combination of two factors to explain the differences in molecular and morphological evolution in penaeids species: i) an accelerated rate of mitochondrial evolution, ii) a slow rate of morphological divergence due to stabilizing selection on morphological or ecological characters. In this study, a unique lineage of *A. antennatus* throughout the study area contrasts sharply with two clearly distinguished monophyletic lineages of *Aristaeomorpha foliacea*.

Before 1920’s there were two recognized species in the genus *Aristaeomorpha*: *A. foliacea* (Risso 1827) from the Mediterranean Sea and Eastern Atlantic, and *A. rostridentata* (Spence Bate 1888) from the Indo-Pacific [62]. Calman [62] compared Spence Bate’s holotype of *A. rostridentata* from Fiji Islands (Pacific Ocean) with *i*) individuals of *A. foliacea* from the Mediterranean Sea and Atlantic coast of Morocco and *ii*) individuals of *A. rostridentata* from the Indian Seas (Arabian Sea, Bay of Bengal and Andaman Sea). Finding no single constant morphological difference between *A. rostridentata* holotype and the individuals of *A. foliacea* from the Mediterranean Sea, Calman [62] observed distinctive morphological differences between *A. rostridentata* holotype, from Pacific Ocean, and the individuals from the Indian Seas. This distinction led him to consider *A. rostridentata* from Fiji Islands as a synonym of *A. foliacea* and to recognize individuals from the Indian Seas as representatives of a distinct species, for which he proposed the name *Aristaeomorpha woodmasoni*, after Wood-Mason, Alcock and Kemp, who had already pointed out some of these morphological differences [62].

The results of Calman’s [62] work, in conjunction with the levels of genetic divergence detected in this study between *A. foliacea* from MED-MOZ and AUS (Figure 2), suggest that cryptic species may have been further overlooked in *A. foliacea* and that allopatric speciation has taken place. The genetic distance between the two monophyletic lineages (*D*_A_ = 0.0226±0.0038) was almost the half of that detected between true congeneric species (Table S3). Furthermore, AUS is distinguished by private NaK genotypes (Table S2) and significant divergence in the frequency of nuclear alleles (Table S6). Calculations of time since divergence based on COI genetic distance indicate that the AUS lineage would have split about 2.88–4.20 Mya (Figure 2b and Table S5). Given that the AUS lineage of *A. foliacea i*) showed strong support from multilocus genealogical concordance, *ii*) inhabits in a recognized distinct biogeographical province (North-Western Australia, [63]) and *iii*) expresses substantial levels of multilocus divergence since the split from a parental lineage, we suggest that *A. foliacea* in NorthWestern Australia should be considered a distinct species. We are confident that if the holotype of *A. rostridentata* from the Fiji Islands, or any paratype, can ever be genetically analyzed, this will result genetically like the AUS lineage of *A. foliacea*. Thus, following the rules of the International Commission on Zoological Nomenclature, we propose to resuscitate *Aristaeomorpha rostridentata* for the Australian lineage. Furthermore, within MED-MOZ lineage *Aristaeomorpha foliacea* expressed monophyletic mitochondrial clusters, with significant divergence in the distribution of allele frequencies (Table S6) that would have diverged about 0.5–0.3 Mya (Figure 1, Figure 2b and Table S5). These two regions (MED and MOZ) can be considered as evolutionary significant units (ESU) according to the definition given by Moritz [64]: “populations that are reciprocally monophyletic for mtDNA alleles and show significant divergence of allele frequencies at nuclear loci”. Therefore each of these species and ESUs should be re-evaluated independently in terms of its potential risk of depletion; and management agencies, e.g. FAO should develop ESU-specific management plans and conservation measures.

### Concluding Remarks

Conservation of biodiversity mostly relies on the taxonomic unit of species as working tool. Therefore, the correct delimitation of species boundaries is essential; yet it is a difficult task that has been an extended focus of discussion since Darwin’s proposal of the morphological species criterion [65]. Sometimes new species have been named without morphological or reproductive evidence, because of the occurrence of cripticity in terms of existence of allopatric monophyletic lineages. Based on genetic evidence, *Aristaeomorpha foliacea* in North-Western Australia would be regarded as a separate and distinct species. We believe that the use of further genes will support the present results; however, an in-depth morphological comparison of the distinct lineages is necessary in order to find diagnosable morphological differences, as has occurred with other penaeids [61]. Finally, *A. foliacea* presently could be considered a cosmopolitan species, as recent records have been recorded in the Western Pacific and Western Atlantic coast [5]. Given the results of this study, we encourage performing combined genetic and morphological analyses throughout the whole distribution range of the species as similar situations of cripticity may arise.

## Supporting Information

Table S1 **Comparison of COI genetic diversity estimates detected in the present and previous works.** Haplotype diversity (*h*), nucleotide diversity (π), standard deviation (S.D.). ALB: Alborán Sea, WM: Western Mediterranean, EM: Eastern Mediterranean, AO: Atlantic Ocean, MOZ: Mozambique Channel, AUS: North-Western Australia.
(DOC)

Table S2 **GenBank accession numbers for each individual analyzed.** COI haplotype number (ch), PEPCK (ph) and NaK (nh) genotype number and allele number. Empty rows correspond to individuals with unsuccessful amplification. The individuals used in phylogenetic analyses are in bold. Location codes are as in Table 1.
(DOC)

Table S3 **Matrix of Tamura-Nei genetic distance measures for concatenated data (1 548 bp) for species and lineages detected in** **Figure 1** **.**
(DOC)

Table S4 **Matrix of genetic distances between all lineages for nuclear genes.** Below diagonal, genetic distances for NaK following Tamura-Nei, and genetic distances for PEPCK are given above diagonal following K2P.
(DOC)

Table S5 **Matrix of Tamura-Nei genetic distances calculated for COI dataset (514 bp) between all lineages (below diagonal) and estimated times (Mya) since divergence (above diagonal), using 0.83–1.2% evolutionary rate (reviewed in Ketmaier et al.** [**51**] **).**
(DOC)

Table S6 **Allele-frequencies homogeneity test for nuclear loci (NaK and PEPCK) between regions for both species.** Contingency chi-squared value (χ^2^), degrees of freedom (df), p-value (*P*).
(DOC)
