# Supplementary material for: Multilocus Comparative Phylogeography of Two Aristeid Shrimps of High Commercial Interest (Aristeus antennatus and Aristaeomorpha foliacea) Reveals Different Responses to Past Environmental Changes
Source: PLoS One. 2013 Mar 13;8(3):e59033. doi: 10.1371/journal.pone.0059033 (PMC3596357; doi:10.1371/journal.pone.0059033)
Supplement: Table S1 — Comparison of COI genetic diversity estimates detected in the present and previous works. Haplotype diversity (h), nucleotide diversity (π), standard deviation (S.D.). ALB: Alborán Sea, WM: Western Mediterranean, EM: Eastern Mediterranean, AO: Atlantic Ocean, MOZ: Mozambique Channel, AUS: North-Western Australia. (DOC) [file pone.0059033.s001.doc]

Table S1. Comparison of COI genetic diversity estimates detected in the present and previous works. Haplotype diversity (*h*), nucleotide diversity (π), standard deviation (S.D.). ALB: Alborán Sea, WM: Western Mediterranean, EM: Eastern Mediterranean, AO: Atlantic Ocean, MOZ: Mozambique Channel, AUS: North-Western Australia.

| *A. antennatus* | Present work | | Fernández et al. [17] | |
| --- | --- | --- | --- | --- |
| Location | *h* ± S.D. | π ± S.D. | *h* ± S.D. | π ± S.D. |
| ALB | 0.533 ± 0.1801 | 0.0029 ± 0.0022 | 0.458 ± 0.085 | 0.0020 ± 0.0005 |
| WM | 0.378 ± 0.1813 | 0.0020 ± 0.0016 | 0.258 ± 0.081 | 0.0014 ± 0.0005 |
| EM | 0.800 ± 0.1001 | 0.0073 ± 0.0045 | 0.758 ± 0.050 | 0.0058 ± 0.0006 |
| AO | 0.911 ± 0.0773 | 0.0052 ± 0.0034 | 0.863 ± 0.042 | 0.0062 ± 0.0008 |
| MOZ | 0.978 ± 0.0540 | 0.0062 ± 0.0040 | 0.961 ± 0.015 | 0.0070 ± 0.0007 |
| *A. foliacea* | Present work |  | Fernández et al. [18] | |
| Location | *h* ± S.D. | π ± S.D. | *h* ± S.D. | π ± S.D. |
| WM | 0.756 ± 0.1295 | 0.0022 ± 0.0017 | 0.649 ± 0.068 | 0.0015 ± 0.0002 |
| EM | 0.644 ± 0.1518 | 0.0032 ± 0.0024 | 0.511 ± 0.081 | 0.0019 ± 0.0003 |
| MOZ | 0.667 ± 0.1633 | 0.0020 ± 0.0016 | 0.557 ± 0.093 | 0.0011 ± 0.0002 |
| AUS | 1.000 ± 0.0447 | 0.0062 ± 0.0034 | 0.990 ± 0.018 | 0.0058 ± 0.0007 |
