# Supplementary material for: Multilocus Comparative Phylogeography of Two Aristeid Shrimps of High Commercial Interest (Aristeus antennatus and Aristaeomorpha foliacea) Reveals Different Responses to Past Environmental Changes
Source: PLoS One. 2013 Mar 13;8(3):e59033. doi: 10.1371/journal.pone.0059033 (PMC3596357; doi:10.1371/journal.pone.0059033)
Supplement: Table S2 — GenBank accession numbers for each individual analyzed. COI haplotype number (ch), PEPCK (ph) and NaK (nh) genotype number and allele number. Empty rows correspond to individuals with unsuccessful amplification. The individuals used in phylogenetic analyses are in bold. Location codes are as in Table 1. (DOC) [file pone.0059033.s002.doc]

Table S2. GenBank accession numbers for each individual analyzed. COI haplotype number (ch), PEPCK (ph) and NaK (nh) genotype number and allele number. Empty rows correspond to individuals with unsuccessful amplification. The individuals used in phylogenetic analyses are in bold. Location codes are as in Table 1.

| Code | | COI (514 bp) | | PEPCK (536 bp) | | | | NaK (498 bp) | | | |
| --- | --- | --- | --- | --- | --- | --- | --- | --- | --- | --- | --- |
| Individual | Location | GenBank | Haplotype | GenBank | Genotype | Allele 1 | Allele 2 | GenBank | Genotype | Allele 1 | Allele 2 |
| Av1 | MOZ | JQ928293 | Av-ch1 | JQ928267 | Av-ph1 | 17 | 18 | JQ928233 | Av-nh1 | 45 | 45 |
| Av2 | MOZ | JQ928294 | Av-ch2 | JQ928267 | Av-ph1 | 17 | 18 | JQ928234 | Av-nh2 | 45 | 46 |
| Av3 | MOZ | JQ928295 | Av-ch3 | JQ928267 | Av-ph1 | 17 | 18 |  |  |  |  |
| Aa1 | ALB | EU908437 | Aa-ch2 | JQ928270 | Aa-ph3 | 1 | 1 | JQ928240 | Aa-nh6 | 3 | 13 |
| Aa2 | ALB | EU908437 | Aa-ch2 | JQ928268 | Aa-ph1 | 3 | 3 | JQ928241 | Aa-nh7 | 21 | 21 |
| Aa3 | ALB | EU908437 | Aa-ch2 |  |  |  |  | JQ928242 | Aa-nh8 | 6 | 27 |
| Aa4 | ALB | EU908437 | Aa-ch2 | JQ928268 | Aa-ph1 | 3 | 3 |  |  |  |  |
| Aa5 | ALB | EU908437 | Aa-ch2 | JQ928268 | Aa-ph1 | 3 | 3 | JQ928243 | Aa-nh9 | 28 | 29 |
| Aa6 | ALB | EU908437 | Aa-ch2 | JQ928268 | Aa-ph1 | 3 | 3 |  |  |  |  |
| Aa7 | ALB | EU908437 | Aa-ch2 |  |  |  |  |  |  |  |  |
| Aa8 | ALB | EU908446 | Aa-ch6 |  |  |  |  |  |  |  |  |
| Aa9 | ALB | EU908497 | Aa-ch14 |  |  |  |  |  |  |  |  |
| Aa10 | ALB | GU972654 | Aa-ch28 | JQ928269 | Aa-ph2 | 3 | 4 | JQ928244 | Aa-nh10 | 11 | 30 |
| Aa11 | WM | EU908437 | Aa-ch2 | JQ928268 | Aa-ph1 | 3 | 3 | JQ928235 | Aa-nh1 | 21 | 22 |
| Aa12 | WM | EU908437 | Aa-ch2 | JQ928269 | Aa-ph2 | 3 | 4 | JQ928236 | Aa-nh2 | 19 | 19 |
| Aa13 | WM | EU908437 | Aa-ch2 | JQ928270 | Aa-ph3 | 1 | 1 |  |  |  |  |
| Aa14 | WM | EU908437 | Aa-ch2 | JQ928271 | Aa-ph4 | 1 | 2 |  |  |  |  |
| Aa15 | WM | EU908437 | Aa-ch2 | JQ928268 | Aa-ph1 | 3 | 3 | JQ928237 | Aa-nh3 | 4 | 13 |
| Aa16 | WM | EU908437 | Aa-ch2 | JQ928268 | Aa-ph1 | 3 | 3 | JQ928238 | Aa-nh4 | 25 | 26 |
| Aa17 | WM | EU908437 | Aa-ch2 | JQ928270 | Aa-ph3 | 1 | 1 |  |  |  |  |
| Aa18 | WM | EU908437 | Aa-ch2 | JQ928269 | Aa-ph2 | 3 | 4 | JQ928239 | Aa-nh5 | 23 | 24 |
| Aa19 | WM | EU908498 | Aa-ch15 | JQ928270 | Aa-ph3 | 1 | 1 |  |  |  |  |
| Aa20 | WM | GU972691 | Aa-ch65 | JQ928269 | Aa-ph2 | 3 | 4 |  |  |  |  |
| Aa21 | EM | EU908437 | Aa-ch2 | JQ928269 | Aa-ph2 | 3 | 4 | JQ928237 | Aa-nh3 | 4 | 13 |
| Aa22 | EM | EU908437 | Aa-ch2 | JQ928272 | Aa-ph5 | 4 | 4 | JQ928245 | Aa-nh11 | 1 | 37 |
| Aa23 | EM | EU908437 | Aa-ch2 | JQ928270 | Aa-ph3 | 1 | 1 | JQ928246 | Aa-nh12 | 35 | 36 |
| Aa24 | EM | EU908437 | Aa-ch2 |  |  |  |  | JQ928247 | Aa-nh13 | 29 | 31 |
| Aa25 | EM | EU908497 | Aa-ch14 | JQ928269 | Aa-ph2 | 3 | 4 |  |  |  |  |
| Aa26 | EM | EU908497 | Aa-ch14 |  |  |  |  | JQ928248 | Aa-nh14 | 1 | 38 |
| Aa27 | EM | EU908497 | Aa-ch14 | JQ928271 | Aa-ph4 | 1 | 2 | JQ928249 | Aa-nh15 | 32 | 32 |
| Aa28 | EM | EU908498 | Aa-ch15 |  |  |  |  |  |  |  |  |
| Aa29 | EM | GU972657 | Aa-ch31 | JQ928269 | Aa-ph2 | 3 | 4 | JQ928250 | Aa-nh16 | 33 | 34 |
| Aa30 | EM | GU972661 | Aa-ch35 | JQ928270 | Aa-ph3 | 1 | 1 |  |  |  |  |
| Aa31 | AO | EU908437 | Aa-ch2 | JQ928271 | Aa-ph4 | 1 | 2 |  |  |  |  |
| Aa32 | AO | EU908437 | Aa-ch2 | JQ928269 | Aa-ph2 | 3 | 4 |  |  |  |  |
| Aa33 | AO | EU908437 | Aa-ch2 | JQ928269 | Aa-ph2 | 3 | 4 |  |  |  |  |
| Aa34 | AO | GU972658 | Aa-ch32 | JQ928270 | Aa-ph3 | 1 | 1 |  |  |  |  |
| Aa35 | AO | GU972658 | Aa-ch32 | JQ928270 | Aa-ph3 | 1 | 1 |  |  |  |  |
| Aa36 | AO | EU908497 | Aa-ch14 | JQ928271 | Aa-ph4 | 1 | 2 | JQ928251 | Aa-nh17 | 3 | 4 |
| Aa37 | AO | EU908498 | Aa-ch15 | JQ928270 | Aa-ph3 | 1 | 1 |  |  |  |  |
| Aa38 | AO | GU972657 | Aa-ch31 | JQ928270 | Aa-ph3 | 1 | 1 |  |  |  |  |
| Aa39 | AO | EU908486 | Aa-ch12 |  |  |  |  | JQ928252 | Aa-nh18 | 1 | 2 |
| Aa40 | AO | EU908539 | Aa-ch24 | JQ928270 | Aa-ph3 | 1 | 1 |  |  |  |  |
| Aa41 | MOZ | GU972658 | Aa-ch32 | JQ928271 | Aa-ph4 | 1 | 2 | JQ928253 | Aa-nh19 | 5 | 6 |
| Aa42 | MOZ | GU972658 | Aa-ch32 | JQ928269 | Aa-ph2 | 3 | 4 | JQ928254 | Aa-nh20 | 11 | 12 |
| Aa43 | MOZ | GU972672 | Aa-ch46 | JQ928269 | Aa-ph2 | 3 | 4 |  |  |  |  |
| Aa44 | MOZ | GU972668 | Aa-ch42 | JQ928271 | Aa-ph4 | 1 | 2 | JQ928255 | Aa-nh21 | 7 | 8 |
| Aa45 | MOZ | GU972682 | Aa-ch56 | JQ928272 | Aa-ph5 | 4 | 4 | JQ928256 | Aa-nh22 | 13 | 14 |
| Aa46 | MOZ | GU972683 | Aa-ch57 | JQ928272 | Aa-ph5 | 4 | 4 | JQ928257 | Aa-nh23 | 15 | 16 |
| Aa47 | MOZ | GU972687 | Aa-ch61 | JQ928270 | Aa-ph3 | 1 | 1 |  |  |  |  |
| Aa48 | MOZ | GU972688 | Aa-ch62 | JQ928269 | Aa-ph2 | 3 | 4 | JQ928258 | Aa-nh24 | 17 | 18 |
| Aa49 | MOZ | EU908437 | Aa-ch2 | JQ928268 | Aa-ph1 | 3 | 3 | JQ928259 | Aa-nh25 | 19 | 20 |
| Aa50 | MOZ | GU972654 | Aa-ch28 | JQ928268 | Aa-ph1 | 3 | 3 | JQ928260 | Aa-nh26 | 9 | 10 |
| Af1 | WM | JN676306 | Af-ch1 | JQ928273 | Af-ph1 | 5 | 5 | JQ928262 | Af-nh2 | 39 | 41 |
| Af2 | WM | JN676306 | Af-ch1 | JQ928274 | Af-ph2 | 11 | 12 |  |  |  |  |
| Af3 | WM | JN676306 | Af-ch1 | JQ928275 | Af-ph3 | 9 | 13 |  |  |  |  |
| Af4 | WM | JN676306 | Af-ch1 | JQ928276 | Af-ph15 | 13 | 14 |  |  |  |  |
| Af5 | WM | JN676306 | Af-ch1 | JQ928273 | Af-ph1 | 5 | 5 | JQ928262 | Af-nh2 | 39 | 41 |
| Af6 | WM | JN676307 | Af-ch2 | JQ928277 | Af-ph5 | 9 | 10 | JQ928263 | Af-nh3 | 41 | 43 |
| Af7 | WM | JN676308 | Af-ch3 | JQ928278 | Af-ph6 | 8 | 9 | JQ928264 | Af-nh4 | 41 | 42 |
| Af8 | WM | JN676308 | Af-ch3 | JQ928279 | Af-ph7 | 13 | 15 | JQ928264 | Af-nh4 | 41 | 42 |
| Af9 | WM | JN676312 | Af-ch7 | JQ928280 | Af-ph8 | 7 | 13 | JQ928262 | Af-nh2 | 39 | 41 |
| Af10 | WM | JN676317 | Af-ch12 | JQ928274 | Af-ph2 | 11 | 12 | JQ928262 | Af-nh2 | 39 | 41 |
| Af11 | EM | JN676306 | Af-ch1 | JQ928281 | Af-ph8 | 7 | 13 | JQ928264 | Af-nh4 | 41 | 42 |
| Af12 | EM | JN676307 | Af-ch2 | JQ928282 | Af-ph10 | 15 | 15 | JQ928264 | Af-nh4 | 41 | 42 |
| Af13 | EM | JN676307 | Af-ch2 | JQ928283 | Af-ph11 | 5 | 6 |  |  |  |  |
| Af14 | EM | JN676307 | Af-ch2 | JQ928284 | Af-ph12 | 5 | 8 | JQ928264 | Af-nh4 | 41 | 42 |
| Af15 | EM | JN676307 | Af-ch2 | JQ928285 | Af-ph13 | 13 | 13 | JQ928264 | Af-nh4 | 41 | 42 |
| Af16 | EM | JN676307 | Af-ch2 | JQ928286 | Af-ph14 | 8 | 8 | JQ928264 | Af-nh4 | 41 | 42 |
| Af17 | EM | JN676307 | Af-ch2 | JQ928287 | Af-ph15 | 13 | 14 |  |  |  |  |
| Af18 | EM | JN676308 | Af-ch3 | JQ928288 | Af-ph16 | 11 | 16 | JQ928264 | Af-nh4 | 41 | 42 |
| Af19 | EM | JN676317 | Af-ch12 | JQ928286 | Af-ph14 | 7 | 15 | JQ928264 | Af-nh4 | 41 | 42 |
| Af20 | EM | JN676317 | Af-ch12 | JQ928290 | Af-ph18 | 7 | 7 | JQ928264 | Af-nh4 | 41 | 42 |
| Af21 | MOZ | JN676331 | Af-ch26 | JQ928290 | Af-ph18 | 7 | 7 | JQ928261 | Af-nh1 | 39 | 40 |
| Af22 | MOZ | JN676331 | Af-ch26 | JQ928286 | Af-ph14 | 8 | 8 | JQ928261 | Af-nh1 | 39 | 40 |
| Af23 | MOZ | JN676331 | Af-ch26 | JQ928278 | Af-ph6 | 8 | 9 | JQ928261 | Af-nh1 | 39 | 40 |
| Af24 | MOZ | JN676331 | Af-ch26 | JQ928283 | Af-ph11 | 5 | 6 | JQ928265 | Af-nh5 | 40 | 42 |
| Af25 | MOZ | JN676331 | Af-ch26 | JQ928277 | Af-ph5 | 9 | 10 | JQ928265 | Af-nh5 | 40 | 42 |
| Af26 | MOZ | JN676331 | Af-ch26 | JQ928286 | Af-ph14 | 8 | 8 | JQ928261 | Af-nh1 | 39 | 40 |
| Af27 | MOZ | JN676332 | Af-ch27 | JQ928286 | Af-ph14 | 8 | 8 | JQ928262 | Af-nh2 | 39 | 41 |
| Af28 | MOZ | JN676336 | Af-ch31 | JQ928277 | Af-ph5 | 9 | 10 | JQ928264 | Af-nh4 | 41 | 42 |
| Af29 | MOZ | JN676334 | Af-ch29 | JQ928286 | Af-ph14 | 8 | 8 | JQ928265 | Af-nh5 | 40 | 42 |
| Af30 | MOZ | JN676335 | Af-ch30 | JQ928291 | Af-ph19 | 8 | 10 | JQ928264 | Af-nh4 | 41 | 42 |
| Af31 | AUS | JN676352 | Af-ch47 |  |  |  |  |  |  |  |  |
| Af32 | AUS | JN676346 | Af-ch41 | JQ928282 | Af-ph10 | 15 | 15 |  |  |  |  |
| Af33 | AUS | JN676356 | Af-ch51 | JQ928286 | Af-ph14 | 8 | 8 | JQ928266 | Af-nh6 | 41 | 44 |
| Af34 | AUS | JN676348 | Af-ch43 | JQ928278 | Af-ph6 | 8 | 9 |  |  |  |  |
| Af35 | AUS | JN676349 | Af-ch44 | JQ928289 | Af-ph17 | 7 | 15 | JQ928266 | Af-nh6 | 41 | 44 |
| Af36 | AUS | JN676350 | Af-ch45 | JQ928291 | Af-ph19 | 8 | 10 | JQ928266 | Af-nh6 | 41 | 44 |
| Af37 | AUS | JN676357 | Af-ch52 | JQ928277 | Af-ph5 | 9 | 10 |  |  |  |  |
| Af38 | AUS | JN676351 | Af-ch46 | JQ928291 | Af-ph19 | 8 | 10 | JQ928266 | Af-nh6 | 41 | 44 |
| Af39 | AUS | JN676358 | Af-ch53 | JQ928292 | Af-ph20 | 10 | 10 | JQ928266 | Af-nh6 | 41 | 44 |
| Af40 | AUS | JN676362 | Af-ch57 | JQ928291 | Af-ph19 | 8 | 10 |  |  |  |  |
