# Supplementary material for: Multilocus Comparative Phylogeography of Two Aristeid Shrimps of High Commercial Interest (Aristeus antennatus and Aristaeomorpha foliacea) Reveals Different Responses to Past Environmental Changes
Source: PLoS One. 2013 Mar 13;8(3):e59033. doi: 10.1371/journal.pone.0059033 (PMC3596357; doi:10.1371/journal.pone.0059033)
Supplement: Table S3 — Matrix of Tamura-Nei genetic distance measures for concatenated data (1 548 bp) for species and lineages detected in Figure 1 . (DOC) [file pone.0059033.s003.doc]

Table S3. Matrix of Tamura-Nei genetic distance measures for concatenated data (1 548 bp) for species and lineages detected in Figure 1.

|  | *A. virilis* | *A. antennatus* | *A. foliacea* | *P. monodon* | Af MED | Af MOZ | Af AUS | Af MED-MOZ |
| --- | --- | --- | --- | --- | --- | --- | --- | --- |
| *A. virilis* | 0.0013 ± 0.00086 |  |  |  |  |  |  |  |
| *A. antennatus* | 0.0492 ± 0.00616 | 0.0033 ± 0.00076 |  |  |  |  |  |  |
| *A. foliacea* | 0.1203 ± 0.01242 | 0.1232 ± 0.01339 | 0.0094 ± 0.00147 |  |  |  |  |  |
| *P. monodon* | 0.2175 ± 0.02067 | 0.2108 ± 0.01854 | 0.1880 ± 0.01709 |  |  |  |  |  |
| *S. crassicornis* | 0.1550 ± 0.01475 | 0.1535 ± 0.01427 | 0.1546 ± 0.01506 | 0.1683 ± 0.01609 |  |  |  |  |
| Af MED | 0.1248 ± 0.01270 | 0.1276 ± 0.01353 |  |  | 0.0016 ± 0.00066 |  |  |  |
| Af MOZ | 0.1248 ± 0.01271 | 0.1254 ± 0.01346 |  |  | 0.0028 ± 0.00132 | 0.0008 ± 0.00035 |  |  |
| Af AUS | 0.1206 ± 0.01228 | 0.1284 ± 0.01326 |  |  | 0.0227 ± 0.00406 | 0.0242 ± 0.00434 | 0.0025 ± 0.00082 |  |
| Af MED-MOZ | 0.1265 ± 0.01307 | 0.1298 ± 0.01316 |  |  |  |  | 0.0226 ± 0.00378 | 0.0030 ± 0.00094 |

*Standard error estimates after 10 000 replicates. In bold are reported within species and within lineage mean values. Location codes as in Table 1.
