# Supplementary material for: Multilocus Comparative Phylogeography of Two Aristeid Shrimps of High Commercial Interest (Aristeus antennatus and Aristaeomorpha foliacea) Reveals Different Responses to Past Environmental Changes
Source: PLoS One. 2013 Mar 13;8(3):e59033. doi: 10.1371/journal.pone.0059033 (PMC3596357; doi:10.1371/journal.pone.0059033)
Supplement: Table S4 — Matrix of genetic distances between all lineages for nuclear genes. Below diagonal, genetic distances for NaK following Tamura-Nei, and genetic distances for PEPCK are given above diagonal following K2P. (DOC) [file pone.0059033.s004.doc]

Table S4. Matrix of genetic distances between all lineages for nuclear genes. Below diagonal, genetic distances for NaK following Tamura-Nei, and genetic distances for PEPCK are given above diagonal following K2P.

|  | *A. virilis* | *A. antennatus* | *A. foliacea* | Af MED | Af MOZ | Af AUS |
| --- | --- | --- | --- | --- | --- | --- |
| *A. virilis* |  | 0.0056 ± 0.0022 | 0.0238 ± 0.0044 | 0.0240 ± 0.0044 | 0.0236 ± 0.0045 | 0.0243 ± 0.0046 |
| *A. antennatus* | 0.0301 ± 0.0064 |  | 0.0214 ± 0.0041 | 0.0217 ± 0.0041 | 0.0213 ± 0.0042 | 0.0219 ± 0.0043 |
| *A. foliacea* | 0.0922 ± 0.0159 | 0.1051 ± 0.0171 |  |  |  |  |
| Af MED | 0.0916 ± 0.0158 | 0.1044 ± 0.0167 |  |  | 0.0005 ± 0.0004 | 0.0001 ± 0.0003 |
| Af MOZ | 0.0926 ± 0.0158 | 0.1055 ± 0.0167 |  | 0.0014 ± 0.0008 |  | 0.0005 ± 0.0001 |
| Af AUS | 0.0963 ± 0.0165 | 0.1094 ± 0.0177 |  | 0.0020 ± 0.0014 | 0.0021 ± 0.0013 |  |
