# Supplementary material for: Multilocus Comparative Phylogeography of Two Aristeid Shrimps of High Commercial Interest (Aristeus antennatus and Aristaeomorpha foliacea) Reveals Different Responses to Past Environmental Changes
Source: PLoS One. 2013 Mar 13;8(3):e59033. doi: 10.1371/journal.pone.0059033 (PMC3596357; doi:10.1371/journal.pone.0059033)
Supplement: Table S5 — Matrix of Tamura-Nei genetic distances calculated for COI dataset (514 bp) between all lineages (below diagonal) and estimated times (Mya) since divergence (above diagonal), using 0.83–1.2% evolutionary rate (reviewed in Ketmaier et al. [51] ). (DOC) [file pone.0059033.s005.doc]

Table S5. Matrix of Tamura-Nei genetic distance calculated for COI dataset (514 bp) between all lineages (below diagonal) and estimated times (Mya) since divergence (above diagonal), using 0.83-1.2% evolutionary rate (reviewed in Ketmaier et al. [51]).

|  | *A. virilis* | *A. antennatus* | *A. foliacea* | *P. monodon* | *S. crassicornis* | Af MED | Af MOZ | Af AUS |
| --- | --- | --- | --- | --- | --- | --- | --- | --- |
| *A. virilis* |  | 7.07-4.87 | 13.40-9.27 | 15.81-10.94 | 12.28-8.49 | 13.40-9.27 | 13.36-9.24 | 12.87-8.90 |
| *A. antennatus* | 0.1143 ± 0.0153 |  | 12.29-8.50 | 15.00-10.37 | 11.71-8.10 | 13.40-9.27 | 13.04-9.02 | 13.63-9.43 |
| *A. foliacea* | 0.1929 ± 0.0194 | 0.1949 ± 0.0186 |  | 13.13-9.08 | 11.29-7.81 |  |  |  |
| *P. monodon* | 0.2625 ± 0.0284 | 0.2489 ± 0.0263 | 0.2137 ± 0.0214 |  | 12.03-8.32 | 14.05-9.72 | 13.62-9.42 | 14.70-10.17 |
| *S. crassicornis* | 0.2038 ± 0.0240 | 0.1944 ± 0.0225 | 0.1874 ± 0.0207 | 0.1997 ± 0.0238 |  | 12.58-8.55 | 12.36-8.55 | 12.39-8.57 |
| Af MED | 0.2224 ± 0.0246 | 0.2225 ± 0.0249 |  | 0.2333 ± 0.0260 | 0.2088 ± 0.0241 |  | 0.54-0.38 | 4.16-2.88 |
| Af MOZ | 0.2217 ± 0.0245 | 0.2165 ± 0.0246 |  | 0.2261 ± 0.0256 | 0.2052 ± 0.0237 | 0.0090 ± 0.0040 |  | 4.20-2.91 |
| Af AUS | 0.2136 ± 0.0236 | 0.2262 ± 0.0252 |  | 0.2441 ± 0.0257 | 0.2056 ± 0.0235 | 0.0690 ± 0.0117 | 0.0698 ± 0.0117 |  |
