# Supplementary material for: Multilocus Comparative Phylogeography of Two Aristeid Shrimps of High Commercial Interest (Aristeus antennatus and Aristaeomorpha foliacea) Reveals Different Responses to Past Environmental Changes
Source: PLoS One. 2013 Mar 13;8(3):e59033. doi: 10.1371/journal.pone.0059033 (PMC3596357; doi:10.1371/journal.pone.0059033)
Supplement: Table S6 — Allele-frequencies homogeneity test for nuclear loci (NaK and PEPCK) between regions for both species. Contingency chi-squared value (χ2), degrees of freedom (df), p-value (P). (DOC) [file pone.0059033.s006.doc]

**Table S6.** Allele-frequencies homogeneity test for nuclear loci (NaK and PEPCK) between regions for both species. Contingency chi-squared value (χ^2^), degrees of freedom (df), p-value (*P*).

|  | NaK |  |  | PEPCK |  |  |
| --- | --- | --- | --- | --- | --- | --- |
|  | χ^2^ | df | *P* | χ^2^ | df | *P* |
| *A. antennatus* |  |  |  |  |  |  |
| MED-ATL | 18.543 | 25 | 0.8185 ^ns^ | 3.034 | 3 | 0.3864 ^ns^ |
| MED-MOZ | 38.894 | 36 | 0.3407 ^ns^ | 9.961 | 3 | 0.0189 ^ns^ |
| ATL-MOZ | 20.000 | 19 | 0.3946 ^ns^ | 9.477 | 3 | 0.0236 ^ns^ |
| *A. foliacea* |  |  |  |  |  |  |
| MED-MOZ | 16.435 | 4 | 0.0025* | 24.930 | 11 | 0.0093* |
| MED-AUS | 20.000 | 4 | 0.0005* | 24.183 | 11 | 0.0119* |
| MOZ-AUS | 21.563 | 4 | 0.0003* | 7.449 | 6 | 0.2813 ^ns^ |

^ns^ non significant, * significant (after Bonferroni correction, based on 0.05 significance level). Location codes as in Table 1.
